# Surface electromyography character of upper limb muscle after open reduction combine with ulnar osteotomy in children with neglected Monteggia fracture

**DOI:** 10.3389/fped.2024.1445385

**Published:** 2024-11-05

**Authors:** Hailing Qiu, Tingzhi Li, Fanling Li, Siqi Zhang, Xiangling Wu, Jing Yang, Xin Li, Ke Fang, Jie Wen, Sheng Xiao

**Affiliations:** ^1^ Department of Pediatric Orthopedics, Hunan Provincial People’s Hospital, The First Affiliated Hospital of Hunan Normal University, Changsha, China; ^2^ Department of Nursing, Hunan Normal University School of Medicine, Changsha, Hunan, China

**Keywords:** children, neglected Monteggia fracture, ulna osteotomy, surface electromyography, muscles of the upper limbs

## Abstract

**Objective:**

This study aims to investigate the surface electromyography (sEMG) characteristics of upper limb muscles in children with neglected Monteggia fracture after open reduction of radial head dislocation and ulna osteotomy and bone grafting internal fixation, and to understand the recovery of muscle activity in children after operation, provide reference for clinical rehabilitation.

**Methods:**

A retrospective analysis was conducted on sixteen children with neglected Monteggia fracture who underwent ulna osteotomy at our hospital from January 2021 to August 2022. The biceps brachii, triceps brachii, flexor carpi radialis, and extensor carpi ulnaris muscle activities were recorded during grip strength tests, flexion and extension of elbow joint while holding a 1 kg dumbbell, as well as gripping tasks. The root mean square (RMS) values of sEMG signals, co-contraction ratio, and elbow joint function scores were compared between pre- and post-operation periods as well as between the affected side and unaffected side.

**Results:**

The preoperative maximum grip strength, as well as the average RMS values of flexor carpi radialis and average RMS value of extensor carpi ulnaris on the affected side were significantly lower. After surgery, both the maximum RMS value of biceps brachii and maximum and average RMS value of extensor carpi ulnaris on the affected side remained lower. Prior to surgery, when performing elbow flexion and extension tests while holding a 1 kg dumbbell, both mean RMS values of biceps brachii and flexor carpi radialis on the affected side were smaller. However, after surgery, there was an increase in mean RMS values of biceps brachii on the affected side. Furthermore, postoperative elbow function scores were significantly higher than preoperative scores.

**Conclusion:**

Open reduction of radial head dislocation combined with ulna osteotomy and bone grafting can achieve good functional activities in the treatment of neglected Monteggia fractures in children. The EMG activity of the extensor carnosus ulnalis muscle on the affected side related to grip strength was low, and the desired effect was not achieved within the expected time.

## 1 Introduction

The Monteggia fracture was initially proposed by the Italian scholar Monteggia in 1814 (1). The traditional definition of a Monteggia fracture refers to a fracture occurring in the middle and upper third of the ulna, accompanied by an anterior dislocation of the radial head. Over time, extensive research has expanded this definition to encompass a composite injury involving multi-directional dislocation of both the radial head and ulna, as well as distal humerus (2). Misdiagnosis of acute Monteggia fracture in children are very common due to the delay and the difficulty in diagnosing, and the missed diagnosis rate can be as high as 28% (3). If not diagnosed early, these lesions can gradually lead to forearm deformities and dysfunction. Including cubitus valgus and osteoarthritis (4), when the course of disease is more than 3 weeks, it eventually evolves into neglected Monteggia fracture.

Surgery is the main treatment for neglected Monteggia fracture in children (5). If not handled properly after operation, it will increase the difficulty of rehabilitation and affect the normal function and development of the elbow joint of children (6). The duration of the disease course for neglected Monteggia fractures ranges from 6 months to 3 years with radial head overgrowth exceeding 2 cm. Commonly employed interventions include proximal ulna osteotomy with lengthening correction and distal radius shortening osteotomy with double plate internal fixation. Oblique ulna osteotomy is primarily utilized to correct ulnar shortening deformities. Prolonged supination plaster fixation for more than 6 weeks following surgery in children may result in complications such as muscle atrophy, functional limitations, and joint stiffness (7, 8). Nevertheless, due to post-surgical bone loss rendering bones fragile, orthopedic surgeons routinely recommend cast removal after 4–6 weeks to prevent re-fracture caused by strenuous activities (9). Reduced compliance towards physical exercise may further contribute to poor adherence towards functional exercises and suboptimal rehabilitation outcomes. Therefore, timely provision of systematic guidance on rehabilitation exercises is crucial for children following plaster removal.

Surface electromyography (sEMG) is a time-series signal of bioelectrical changes during neuromuscular system activity recorded on the surface of muscles (10). It provides real-time and accurate reflection of muscle activity, function, and fatigue state without causing damage. The utilization of sEMG for assessing muscle function has emerged as a significant area in neurorehabilitation medicine research in recent years (11). Changes in sEMG signals are closely associated with central control and the physiological processes within muscles, enabling the study of active and antagonistic muscle activity during motor control. Consequently, monitoring alterations in sEMG signals not only contributes to understanding neural control mechanisms among stroke patients (12), but also serves as an important tool for evaluating muscle spasticity and rehabilitation efficacy in affected limbs (13, 14). Additionally, it can predict factors such as muscle fiber type, activation time, force generation sequence, strength levels etc (15–17). The RMS of sEMG signal can reflect neuromuscular function, and is positively correlated with muscle strength and muscle tone in active and passive contraction, respectively (18), which can be used to analyze the recruitment of muscle fibers during muscle contraction (19).

Currently, the majority of studies on neglected Monteggia fractures in children primarily focus on evaluating treatment efficacy and functional outcomes based on clinical manifestations and imaging (20–22). However, there is limited research investigating muscle activity recovery in children after surgery, leaving uncertainty regarding the timeliness and effectiveness of postoperative rehabilitation training. This study aims to employ surface electromyography to assess postoperative muscle activity in children with neglected Monteggia fractures. The findings will enhance understanding among pediatric orthopedic surgeons regarding micro-recovery of muscle activity following surgery for neglected Monteggia fractures in children, thereby providing valuable insights for clinical rehabilitation.

## 2 Methods

A total of 16 children with previous Monteggia fractures, who were admitted to the department of Pediatric Orthopedics at one provincial tertiary hospital between January 2021 and August 2022, were included in this study. The cohort consisted of 12 males and 4 females, with an average age of (9.13 ± 3.12) years, height of (1.40 ± 0.21) m, and weight of (33.76 ± 13.43) kg. The mean BMI index was (16.48 ± 2.33). Among the patients, the left hand was affected in 10 cases while the right hand was affected in 6 cases; specifically, there were 15 right-dominant hands and one left-dominnant hand observed among them. This study received approval from the local ethics committee and informed consent forms were signed by all family members involved.

Inclusion criteria: (1). Age range of participants was between 5 and 15 years old. (2). Surgical procedures included open reduction of radial head dislocation, ulna osteotomy, bone grafting, and internal fixation. (3). The affected limb has been removed from the external cast fixation and has resumed normal activities, but the internal fixation of the ulna has not been removed. (4). Throughout the study period, no other conditions affecting motor function such as muscle strain or sprain were present except for this fracture case. (5). No vigorous exercise that affected muscle activity was performed within 48 h.

Exclusion criteria: (1). Exclusion of fresh Monteggia fracture, multiple fractures, chronic or infectious diseases affecting bone metabolism and fracture healing. (2). Exclusion of pathological fractures combined with other bone diseases and bone tumors; 3. Exclusion of individuals with intellectual disability who are unable to follow instructions to complete the prescribed movements; Additionally, exclusion of those with upper limb nerve injury or neuromuscular disease.

All patients underwent open reduction of radial head dislocation and ulna osteotomy with bone graft and plate internal fixation under general anesthesia, as well as annular ligament reconstruction when necessary. All procedures were performed by associate professors of pediatric orthopedics. The surgical procedures were performed using the Body intermuscular approach. During the operation, utmost attention was given to safeguarding the flexor digitorum profundus and extensor carpi ulnaris muscles from transverse cutting or fiber injury. Professional postoperative rehabilitation exercises were administered, preceded by a detailed explanation of the functional exercise process to parents for their cooperation in achieving exercise goals. Before the operation, all the children received education and video watching of upper limb related functional exercise, and guided them to exercise, twice a day, 10–15 min each time. Our functional rehabilitation protocol consisted of four stages that strictly adhered to principles of progressive intensity and stepwise progression.

In the initial phase, immediate strengthening of forearm muscle contraction was observed upon emergence from anesthesia. Isometric contractions were performed on fixed segments, while isotonic contractions were executed on unfixed joints such as finger flexion and extension, as well as fist clenching exercises. Each exercise was repeated 20 times in three sets per day, with a focus on achieving full range of motion during finger extension and fist clenching. In cases where cooperation was limited, a combination of active and passive movements was employed; however, passive movements served solely as auxiliary training when independent movement or cooperation was not feasible. Additionally, gentle movements including massage and assisted joint flexion-extension were implemented to avoid inducing moderate to severe pain. During the subsequent stage following reduction and fixation (1–2 weeks post-surgery), effective muscle contraction exercises continued alongside increased shoulder joint mobility for 10–15 min twice daily. In the third stage (3–4 weeks after surgery), exercise duration and frequency were augmented if deemed appropriate—three times daily for 15–30 min each session. Finally, during the clinical healing period (4 to 6 weeks post-surgery) after cast removal, emphasis shifted towards restoring elbow joint flexion-extension and forearm rotation through three sessions lasting 15–30 min per day. During hospitalization, the patient was reminded to take functional exercise every day, and the functional exercise was recorded in nursing documents. After discharge, the patient was followed up at the outpatient department, and the results were recorded in the outpatient medical record.

### 2.1 Measurement of outcome

#### 2.1.1 Mayo elbow function score

The Mayo elbow function score was utilized to assess the elbow function of pediatric patients, with a total possible score of 100 points, consisting of 45 points for pain, 20 points for range of motion, 10 points for stability, and 25 points for activities of daily living. Scores below 60 were considered poor, scores between 60 and 74 were deemed fair, scores between 75 and 89 were classified as good, while scores ranging from 90 to100 were rated as excellent. The rate of excellent or good outcomes was calculated by dividing the sum of excellent cases plus good cases by the total number of cases and multiplying this quotient by one hundred percent.

#### 2.1.2 Grip strength test

Participants were positioned in an upright chair, ensuring that their feet were flat on the floor and their elbows rested straight on the table. Their forearm was maintained in a neutral position with the wrist extended neutrally. Holding a calibrated electronic grip strength tester (brand: CAMRY, model: EH101, MAX: 90 kg, d = 100 g) for optimal grip, the child underwent three trials of the test, each lasting 5–7 s with a 1-minute interval between them. The maximum reading obtained from these trials was selected for subsequent analysis. For participants unfamiliar with the handgrip dynamometer, an assessor provided a demonstration prior to testing by gently gripping it and observing numerical changes displayed electronically to familiarize them with the procedure. During the grip strength assessment, simultaneous collection of surface electromyography signals from both flexor carpi radialis (forearm flexor) and extensor carpi ulna (forearm extensor) muscles occurred at maximal grip strength levels for further analysis purposes. Analysis software associated with surface electromyography testing system was employed to calculate RMS maximum value, RMS mean value and Integrate electromyography (iEMG) mean value. Additionally, co-contraction ratio of extensor carpi ulna = extensor carpi ulna iEMG/(extensor carpi ulna iEMG + flexor carpi radialis iEMG) was determined based on iEMG measurements.

#### 2.1.3 1 kg dumbbell elbow flexion and extension test

**Figure 1:**
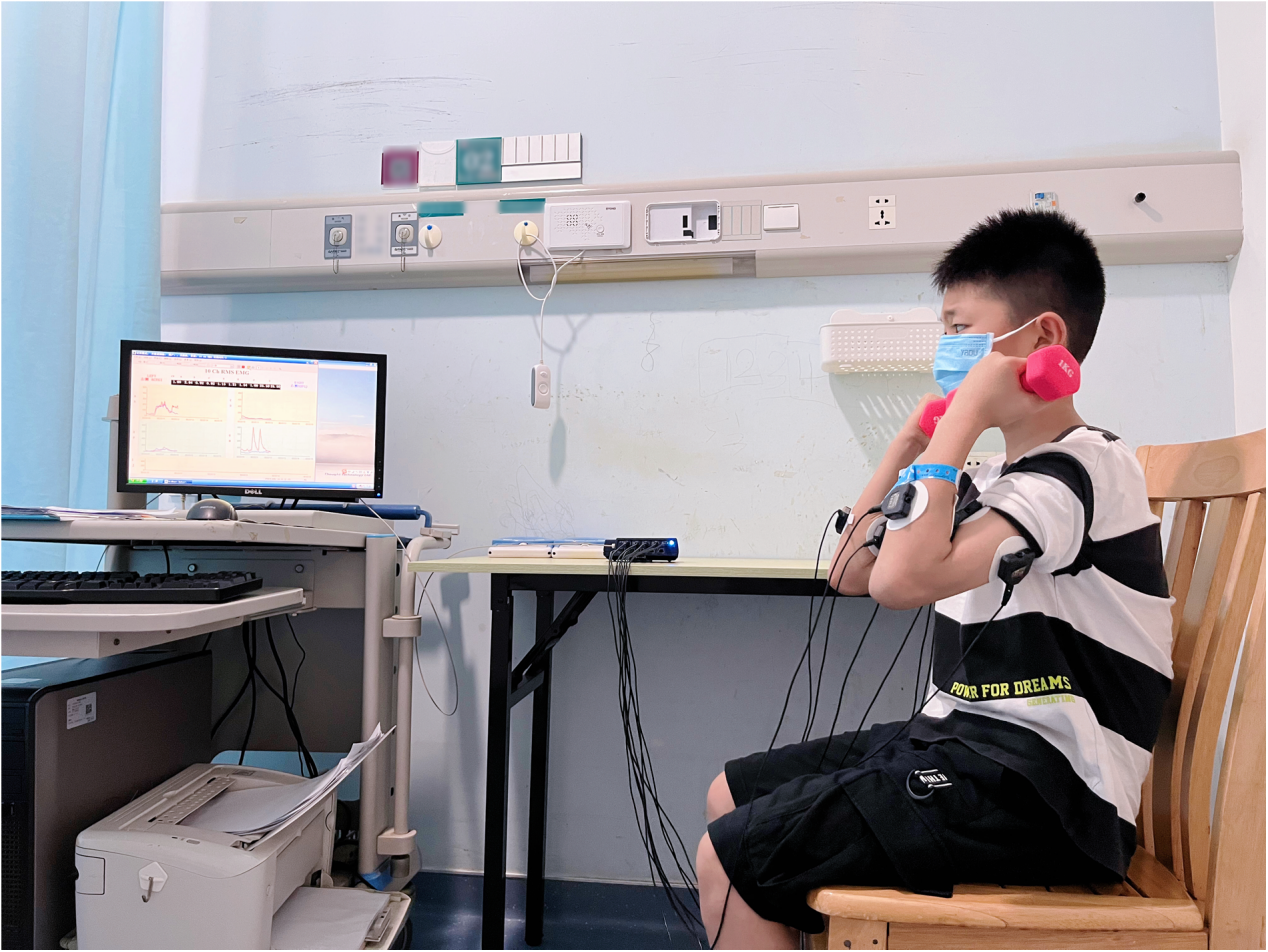
Surface electromyography test of the child holding a 1 kg dumbbell.

Participants were seated on a straight-backed chair with their feet flat on the floor, gripping a dumbbell in hand with their fist facing upwards (Figure 1). The dumbbell is then lifted upwards, requiring maximum flexion and extension of the range of motion. Each movement is performed for 8 repetitions per set, with 4–6 sets completed. To eliminate any extraneous interference from upper limb movements at the beginning of the activity, the waveforms of the first two flexion and extension movements as well as the last flexion and extension movements were excluded. Surface electromyography signals were synchronously collected from biceps brachii, triceps brachii, flexor carpi radialis, and extensor carpi ulnalis muscles. The system automatically calculated mean RMS values and mean integrated electromyography (iEMG). The cocontraction ratio was determined using iEMG values: Triceps cocontraction ratio = triceps iEMG/(triceps iEMG + biceps iEMG).

#### 2.1.4 Grip egg grip

Participants were seated on a straight-backed chair with their feet flat on the floor. The elbow joint is positioned straight on the table, while the forearm remains in a neutral position and the wrist is extended neutrally. During hand practice for pinch force, participants grip an egg-shaped plastic ball in their palm using all five fingers except for the thumb, gradually releasing it after 5 s of grasping. Electromyography (EMG) measurements of flexor carpi radialis and extensor carpi ulnaris are taken three times with approximately 2 min intervals. The maximum peak EMG value is selected for analysis, while simultaneously monitoring surface EMG data of flexor carpi radialis and extensor carpi ulnaris.

#### 2.1.5 Surface electromyography test

The surface electromyography tests were conducted in a controlled environment at 26 ± 2℃ within the designated room, all the sEMGs tests were performed by the same technician. A round, disposable Triodes dry electrode (5.6 cm peripheral diameter, 1.0 cm electrode diameter) was utilized as the test electrode. The recording electrode was positioned at a distance of 2 cm from the center of the reference electrode. Both upper limbs of the subjects were fully exposed and their skin was thoroughly cleansed with 75% medical alcohol to remove any residual oils or impurities. The electrodes were carefully placed parallel to the long axis of muscle fibers in the biceps, triceps, flexor carpi radialis, and extensor carpi ulnalis muscles for accurate signal collection during grip strength tests, elbow flexion and extension holding of a 1 kg dumbbell, as well as grip egg grasping tasks. Prior to testing, clear explanations and demonstrations were provided to ensure understanding among participants. Subsequently, mean RMS value, maximum RMS value and iEMG value data were processed using specialized equipment's data processing system.

SEMG was performed before operation, 8 weeks and 6 months after operation, and before the ulna internal fixation was removed. The sEMG data before operation and at the last follow-up were analyzed.

### 2.2 Statistical analysis

All data in accordance with normal distribution were expressed as (x ± s), and the results of measurement data in non-normal distribution were expressed as median and quartile. The data were analyzed using SPSS 26.0 statistical software. Analysis was conducted on the data collected before the operation and at the last follow-up. In this study, the d value of preoperative and postoperative Mayo elbow joint function scores of children was in line with normal distribution, so paired *T*-test was used. The values of maximum grip strength, mean RMS, maximum RMS and difference d of surface electromyography signal of biceps, triceps, flexor carpi radialis and extensor carpi ulna in all children did not conform to normal distribution, so nonparametric test was used. Specifically, Wilcoxon signed-rank test was used to compare preoperative and postoperative data as well as affected side vs. unaffected side with a significance level set at *α* = 0.05.

## 3 Results

The patients were followed up for an average duration of (11.06 ± 4.12) months. The preoperative Mayo score of the affected elbow was 80.31 ± 3.40, with an excellent and good rate of 85%. At the final follow-up, the Mayo score of the elbow joint improved to 96.25 ± 2.89, with a remarkable excellent and good rate of 100%, demonstrating a significant difference compared to preoperative values (*p* < 0.05) (Table 1).

**Table 1:** Results of mayo elbow joint function score before and at the last postoperative follow-up.

Group	Mean ± SD	*d* value and 95% CI	*t*-test	
			*t*-value	*p*-value
Pre-OP	80.31 ± 3.40	1.04 (13.7 ± 18.1)	15.29	0.01
Post-OP	96.25 ± 2.89			

In the grip strength test, the preoperative maximum grip strength value, the average RMS value of flexor carpi radialis and the maximum and average RMS value of extensor carpi ulnaris of the affected side were lower than those of the unaffected side (*p* < 0.05). The maximum RMS value of biceps brachii and the maximum and average RMS value of extensor carpi ulnaris on the affected side were smaller than those on the unaffected side (*p* < 0.05) (Table 2).

**Table 2:** Comparison of grip strength test results.

	Affected side Pre-OP	Unaffected side Pre-OP	Affected side Post-OP	Unaffected side Post-OP
Maximum grip strength	11.35 (6.23, 19.40)*	12.10 (8.08, 21.85)	13.50 (6.63, 20.85)	13.50 (8.35, 21.65)
Maximum RMS value of biceps	226.82 (142.04, 317.79)	254.27 (156.52, 366.94)	219.63 (167.28, 346.22,)**	297.52 (216.93, 372.88)
Maximum RMS value of triceps	143.73 (117.50, 207.53)	161.32 (104.92, 252.12)	173.44 (118.19, 255.21)	184.79 (153. 00, 309.81)
Maximum RMS value of flexor carpi radialis	267.14 (230.33, 405.15)	301.53 (243.29, 412.89)	274.55 (228.57, 412.22)	310.24 (258.09, 348.23)
Maximum RMS value of extensor carpi ulnar	274.83 (241.33, 342.20)*	308.74 (263.57,364.35)	250.52 (223.50, 419.79)**	292.10 (249.79, 480.88)
Mean RMS value of biceps	113.74 (71.38, 158.64)	133.03 (70.39, 164.26)	116.80 (69.45, 171.20)	149.45 (108.44, 178.04)
Mean RMS value of triceps	82.19 (62.30, 104.50)	80.76 (58.19, 130.43)	88.48 (54.49, 138.59)	116.65 (65.06,150.03
Mean RMS value of flexor carpi radialis	167.33 (152.58, 254.38)*	206.82 (153.32, 277.84)	178.74 (145.77, 262.26)	181.37 (161.11, 242.08)
Mean RMS value of extensor carpi ulnar	158.27 (147.60, 237.65)*	197.50 (172.17, 255.94)	163.12 (138.93, 267.27)**	246.95 (158.40, 289.41)
Co-contraction ratio of triceps	0.41 (0.36,0.52)	0.44 (0.35, 0.48)	0.44 (0.35, 0.62)	0.43 (0.38, 0.51)
Co-contraction ratio of extensor carpi ulnar	0.50 (0.42, 0.56)	0.54 (0.44, 0.58)	0.50 (0.44,0.55)	0.52(0.49, 0.60)

* Represents the comparison between the affected side and the unaffected side before surgery, *p* < 0.05.

** Represents the comparison between the postoperative affected side and the postoperative unaffected side, *p* < 0.05.

In the 1 kg dumbbell elbow flexion and extension test, the preoperative mean RMS values of biceps brachii and FCR on the affected side were smaller than those on the unaffected side (*p* < 0.05). The mean RMS of biceps brachii on the affected side before operation was smaller than that on the affected side after operation (*p* < 0.05) (Table 3).

**Table 3:** Comparison of test results of 1 kg dumbbell flexion elbow.

	Affected side pre-OP	Unaffected side pre-OP	Affected side post-OP	Unaffected side post-OP
Mean RMS value of biceps	168.94 (150.00, 218.30)*	227.36 (166.78, 289.04)	234.63 (196.39, 258.50)**	229.48 (168.29, 306.59)
Mean RMS value of triceps	51.59 (34.72, 70.00)	59.65 (46.02, 67.44)	60.10 (46.33, 77.71)	57.18 (50.78, 68.81)
Mean RMS value of flexor carpi radialis	107.38 (70.25, 138.07)*	135.50 (113.50, 153.39)	111.92 (91.47, 133.46)	120.89 (90.19, 136.83)
Mean RMS value of extensor carpi ulnar	74.43 (49.48, 94.65)	81.23 (64.00, 97.41)	76.88 (59.41, 94.06)	81.08 (67. 00, 88.72)
co-contraction ratio of triceps	0.23 (0.16, 0.27)	0.19 (0.17, 0.28)	0.20 (0.17, 0.29)	0.20 (0.15, 0.27)
Co-contraction ratio of extensor carpi ulnar	0.44 (0.38, 0.53)	0.41 (0.35, 0.44)	0.43 (0.36, 0.50)	0.43 (0.37, 0.45)

* Represents the comparison between the affected side and the unaffected side before surgery, *p* < 0.05.

** Represents the comparison between the affected side pre-operation and the affected side post-operation, *p* < 0.05. Data were expressed as median(25% quartile, 75% quartile).

In the grip egg test, there was no significant difference in the mean RMS and cocontraction ratio of the measured site before and after operation, unaffected side and affected side (*p* > 0.05) (Table 4).

**Table 4:** Comparison of the values of grip eggs.

	Affected side pre-OP	Unaffected side pre-OP	Affected side post-OP	Unaffected side post-OP
Mean RMS value of biceps	98.15 (50.32, 175.34)	115.53 (63.94, 221.06)	106.11 (66.87, 197.53)	131.70 (67.19, 224.87)
Mean RMS value of triceps	61.26 (38.45, 125.00)	67.81 (42.52, 85.36)	66.01 (42.12, 125.85)	63.85 (55.24, 177.84)
Mean RMS value of flexor carpi radialis	157.00 (100.24, 173.44)	175.75 (144.05, 207.64)	153.24 (118.23, 217.38)	159.28 (138.19, 174.29)
Mean RMS value of extensor carpi ulnar	164.10 (126.25, 213.03)	188.02 (133.22, 279.65)	196.86 (147.13, 243.13)	205.23 (128.46, 245.17)
Co-contraction ratio of triceps	0.38 (0.28, 0.51)	0.37 (0.30/0.50)	0.42 (0.28, 0.51)	0.40 (0.35, 0.46)
Co-contraction ratio of extensor carpi ulnar	0.53 (0.47, 0.59)	0.51 (0.47, 0.58)	0.53 (0.48, 0.58)	0.51 (0.50, 0.59)

## 4 Discussion

The Neglected Monteggia fracture is primarily characterized by cubitus valgus and limited flexion. Prolonged malunion of the ulna results in contracture of the interosseous membrane at the fracture site, leading to restricted forearm rotation. Following a forearm injury, children often maintain a supination position for an extended period, which can result in pronation dysfunction. In this study, all enrolled children underwent treatment using the Body intermuscular approach, specifically entering through the intermuscular space between the extensor carpi ulnaris and flexor digitorum profundus muscles. Special attention was given to muscle protection during surgery to avoid transverse cutting or damage to muscle fibers, ensuring rapid and effective postoperative muscle rehabilitation.

Grip strength is primarily generated by the flexors of the forearm (23). Following upper limb fractures in children, a decrease in grip strength is commonly observed and serves as an important indicator for assessing hand function impairment and treatment efficacy (24). Pershad et al. (25) discovered that grip strength in the affected hand may decrease by more than 20% compared to the healthy hand in children with forearm fractures. Hepping AM et al. (26) reported that at 6 weeks, 3 months, and 6 months post-fracture, strength loss in the affected hand was measured at 32.3%, 12.8%, and 4.7% respectively when compared to the healthy hand among children and adolescents. However, another study (27) found that after an average follow-up period of 8.4 months following internal fixation of ulna and radius fractures in children, grip strength on the affected side increased while grip strength on the healthy side decreased correspondingly until both sides reached equivalent levels post-surgery. Consistent with previous findings, maximum grip strength on the affected side was significantly lower than that of the healthy side prior to surgery; however, no difference between both sides was observed after surgery.

The activation of forearm muscles plays a crucial role in grip stability generation (28). Pathological changes in the affected forearm, including malunion, radial head dislocation, and soft tissue injury, have resulted in diminished muscle conduction strength. In the grip strength test conducted for this study, the maximum and average RMS values of the extensor carpi ulnaris and RMS values of flexor carpi radialis on the affected side were lower than those observed on the unaffected side prior to surgery. This finding aligns with the clinicopathological manifestations associated with neglected Monteggia fracture.

For children with neglected Monteggia fracture, the elbow joint was immobilized at 90° of flexion using a plaster cast, while the forearm was positioned in neutral or supination at 30°. After a period of 4 weeks, the plaster cast was removed and functional exercises were initiated (29). The postoperative plaster fixation resulted in limited joint mobility and prolonged skeletal muscle inactivity, leading to muscle protein loss and subsequent fiber atrophy (30). A study conducted on adults with distal radius fractures found that hand immobilization for 6 weeks led to decreased intrinsic compound muscle action potential amplitude and significant reduction in hand muscle fiber area (31). Disuse muscle atrophy has both macroscopic and microscopic effects on muscles, characterized by reduced endurance, strength, as well as decreased muscle fiber area and volume. In this study, it was observed that the maximum and average RMS values of extensor carpi ulnalis on the affected side were smaller compared to those of the unaffected side; similarly, the maximum RMS value of biceps brachii was smaller than that of the unaffected side. It should be noted that in this study, the maximum RMS value of the biceps brachii on the affected side was smaller than that on the unaffected side in the grip strength test after surgery. This result cannot indicate that the EMG activity of the biceps brachii on the affected side has not yet recovered, because the maximum RMS value of the biceps brachii on the unaffected side increased significantly after surgery, but the maximum RMS value of the biceps brachii on the affected side did not change significantly before and after surgery.

A study (32) found that the grip strength and hand muscle working ability of patients with closed radial fractures decreased significantly at 1 month after surgery, but gradually recovered after 2 months. The hand muscle activity of the child should be consistent with the results of the grip strength test, and there is no difference between the unaffected sides. However, we speculate that the discrepancy between grip strength and EMG results after the operation in this study may be due to the increased dependence of the children due to the higher attention they received in daily life when the affected side was injured and after the operation. At the same time, caregivers are more inclined to compromise and give up when they do not cooperate due to pain and other discomfort during functional exercise (33), resulting in insufficient rehabilitation training of the affected limb of the child, poor recovery of muscle strength of the affected forearm, and low muscle strength. During the maximum work task, the posterior group of forearm muscles were less activated, and the electromyography activity of extensor carpi ulna was less. The physiological and psychological factors of the child and the overprotection of the family members will also make the child rely on the unaffected hand for a long time, and even lead to the change of the dominant hand. This study indeed showed that the dominant hand of the child changed.

Elbow flexion and extension activities are crucial for children's daily functioning, with the biceps brachii muscle group being identified as the primary contributor to elbow flexion and supination (34). In this study, the mean RMS values of both the biceps brachii and flexor carpi radialis muscles were found to be smaller before surgery compared to those of the unaffected side during a 1 kg dumbbell elbow flexion and extension test. This finding aligns with previous results from grip strength tests conducted prior to surgical intervention for neglected Monteggia fractures. Disuse atrophy resulting from immobilization leads to rapid loss of muscle mass and strength; however, active training can counteract these effects by restoring muscle strength (35, 36). Over time, upper limb function gradually improves in children. Our study demonstrated a significant increase in electromyography (EMG) activity of both the biceps and triceps on the affected side after surgery, with no difference observed between the affected side and unaffected side. These results indicate that surgical intervention can restore normal anatomical alignment of the brachial-radial joint, correct ulnar deformity, and maximize restoration of elbow joint flexion-extension function. Furthermore, based on preoperative and postoperative Mayo elbow function scores for the affected side, our findings demonstrate favorable functional recovery outcomes following surgical treatment for neglected Monteggia fractures.

Paradoxically, this study revealed no discernible difference in semG activity of the measured muscles before and after the operation, both on the affected and unaffected sides of the children during power egg grasp. However, these findings were inconsistent with those obtained from handgrip dynamometer tests. Despite similarities between these two test movements, potential reasons for such inconsistency may include the soft material and larger grip volume of the grip egg compared to that of the dynamometer. Additionally, it is possible that forearm muscles are not fully activated when grasping the grip egg. In comparison to grip tests, force exerted by gripping an egg is smaller and requires less work from forearm muscles. Consequently, EMG activity levels remained similar before and after surgery. This conjecture was partially supported by lower average RMS values observed during grip egg grasp compared to those recorded during grip dynamometer tests. Furthermore, results from grip strength assessments indicated that muscle function in the affected limb was sufficient for performing everyday tasks or activities requiring minimal effort.

The co-contraction ratio (CR) refers to the proportion of antagonistic muscle contraction during active muscle contraction. There were no significant differences observed in the cocontraction ratios of the triceps brachii and extensor carpi ulna between the unaffected and affected sides before and after surgery, suggesting that the neuromuscular innervation in children participating in this study was normal, and motor control did not affect the activity of both active and antagonist muscles.

In this study, electromyography analysis during grip strength testing revealed that after an average follow-up of 11 months, patients with neglected Monteggia fractures exhibited lower electromyography activity in the extensor carpi ulnaris compared to their unaffected side. This finding provides a reference for designing rehabilitation exercise programs following internal fixation removal. Combining aerobic and resistance exercises with child-weight dumbbells and barbell programs can improve muscle strength and mass in the affected forearm. Gamification is a novel intervention for functional recovery and rehabilitation. Previous studies (37, 38) have demonstrated positive outcomes when using video games combined with conventional treatment or immersive virtual games to treat upper limb fractures, including pain relief, improved elbow and wrist function, increased joint range of motion (ROM), enhanced patient compliance, and increased grip strength. Neuromuscular electrical stimulation (NMES) has also been recommended as it has been shown to increase skeletal muscle mass in both young and older adults (39). Additionally, mental exercises can prevent loss of hand function associated with immobilization over the medium- to long-term period; its application in upper limb orthopedic rehabilitation has achieved expected clinical results (40, 41).

The limitations of this study are that the sEMG test has a large error and certain limitations, which may not be able to reasonably explain the differences in the unaffected limb when tested. For example, there is a difference in the maximum RMS value of the postoperative biceps brachii on the affected side and the unaffected side during the grip strength test. This is due to the significant increase in the maximum RMS value of the unaffected side of the biceps brachii muscle after surgery. Conventionally, the surface electromyography value of the unaffected side should not change significantly before and after surgery. Secondly, the sample size of this study is small and it is an exploratory study, and future research should include multi-center, large-sample prospective studies to obtain evidence from evidence-based medicine. In the future, we plan to investigate the changes in lifestyle and psychological state of children after surgery and explore rehabilitation exercise methods that are more suitable for children, which may be related to the rehabilitation effect of children. There are two ways to obtain more accurate data in subsequent research: first, to find a more suitable test method for children; The second is to select more accurate and sensitive EMG electrodes.

## 5 Conclusion

In conclusion, open reduction of radial head dislocation combined with ulna osteotomy and bone grafting can achieve good functional activities in the treatment of neglected Monteggia fractures in children. However, the EMG activity of the extensor carnosus ulnalis muscle on the affected side related to grip strength was low, and the desired effect was not achieved within the expected time. These findings suggest that pediatric orthopedist should provide timely rehabilitation intervention and guidance for the rehabilitation of the posterior forearm muscles of children to maintain the stability of wrist function.

## Data Availability

The raw data supporting the conclusions of this article will be made available by the authors, without undue reservation.
